# Constructing Polyimide Aerogels with Carboxyl for CO_2_ Adsorption

**DOI:** 10.3390/polym14030359

**Published:** 2022-01-18

**Authors:** Yangfeng Gao, Chao Dong, Fan Zhang, Hongwei Ma, Yang Li

**Affiliations:** 1 State Key Laboratory of Fine Chemicals, Dalian University of Technology, 2 Linggong Road, Dalian 116024, China; gyf112@126.com (Y.G.); dongchao@dlut.edu.cn (C.D.); 2 Weifang Hongrun New Materials Co., Ltd., Weifang 261108, China; zhangfan200801073@163.com

**Keywords:** polyimide aerogels, CO_2_ adsorption, carboxyl groups, mesoporous

## Abstract

In this study, mesoporous polyimide aerogels with carboxyl were successfully synthesized by the co-polymerization method at room temperature from pyromellitic dianhydride and 1,3,5-triaminophenoxybenzene, 3,5-diaminobenzoic acid, and 2,2′-dimethyl-4,4′-diaminobiphenyl. Compared to previously reported porous organic polymer materials, this aerogel has the advantage of a simple and efficient synthesis method. The thermal decomposition temperatures of the obtained polyimide aerogels are all above 400 °C and have excellent thermal stability. Among them, the largest specific surface area is 62.03 m^2^/g. Although the surface area of this aerogel is not large enough, it has considerable CO_2_ adsorption properties. The adsorption capacity of CO_2_ is up to 11.9 cm^3^/g, which is comparable to those of previously reported porous materials. The high CO_2_ adsorption is attributed to the abundance of carboxyl groups in the polyimide networks. The mild and convenient synthesis method and high CO_2_ adsorption capacity indicate that the polyimide aerogel with carboxyl is suitable as a good candidate material for CO_2_ adsorption.

## 1. Introduction

Carbon dioxide is considered the main cause of the greenhouse effect. The excessive emissions of CO_2_ are not only contributing to global warming, but also to rising sea levels. In order to solve this environmental problem, scientists have conducted a great deal of research. In recent years, the development of porous organic polymer materials based on CO_2_ adsorption, such as metal organic frameworks (MOFs) [1,2,3,4], covalent organic frameworks (COFs) [5,6,7,8], conjugated microporous polymers (CMPs) [9,10,11,12], etc., has received considerable attention. Although these porous materials have high CO_2_ adsorption properties, their synthesis process is extremely complicated and the reaction conditions are harsh, making it difficult to achieve large-scale production and application. Therefore, it is highly desirable to develop efficient, low-cost, and recyclable solid sorbent materials for CO_2_ capture.

Aerogel is an ultra-light porous organic material with low density, high surface area, a low dielectric constant, and low thermal conductivity [13,14]. Due to its superior performance, it has attracted intense interest and has been widely applied to thermal insulation, acoustic insulation [15], electrodes for lithium-ion batteries [16,17], and catalysis [18]. By supercritical fluid extraction or freeze-drying, aerogel can be efficiently obtained to maintain the porosity of the solid network structure. Among various aerogels, silica aerogel [19] is the most well-known and extensively studied since it was first synthesized in the 1930s. It demonstrates excellent thermal insulation performance and has been utilized in the aerospace field for collecting comet dust particles in the NASA Stardust Mission and as insulation materials for the batteries and electronics on the Mars Rover [20]. Despite silica aerogel being a promising material, its applications are restricted by its poor mechanical properties and fragile nature [21]. Hence, numerous studies have been conducted to improve its mechanical properties via modification. Experimental results suggest that reacting oligomers such as melamine-formaldehyde [22], epoxy [23], styrene [24], and cellulose [25] with function groups (hydroxyl, amine or vinyl) on the silica surface can greatly improve the mechanical properties of silica aerogels and consistently endow them with several characteristics such as super-hydrophobicity, adsorptivity, and flexibility. Nevertheless, the use temperature of these polymer-reinforced silica aerogels is usually below 200 °C, which indicates that the materials have relatively weak thermal stability.

Organic polymer aerogels are solid porous materials that have been developed relatively rapidly in recent years, especially polyimide aerogels. Polyimide aerogels having high porosity, low thermal conductivity, flexibility, and low density have become a popular research topic in the field of polyimide materials. Although the specific surface area of polyimide aerogels is not as high as that of previously reported microporous materials, they have the advantages of a facile synthesis method, short reaction time, safe operation process, and of being environmentally friendly. In the past few years, polyimide aerogels have been successfully fabricated through crosslinking amine-terminated or anhydride-terminated polyimide wet gels, and dried by supercritical CO_2_ drying [26,27] or the freeze-drying [28] process. Meador et al. [29], from the NASA Glenn Research Center, systematically investigated the preparation of polyimide aerogels crosslinked with 1,3,5-triaminophenoxybenze (TAB) and1,3,5-benzenetricarbonyl trichloride (BTC), separately. Shen et al. [30,31] fabricated a low dielectric constant polyimide aerogel that used oct(aminophenyl) silsesquioxane (OAPS) as the crosslinking agent and a highly thermally resistant and flexible polyimide aerogels crosslinked with 2,4,6-tris(4-aminophenyl)pyridine (TAPP). Superior nanoporous polyimide aerogels crosslinked with 1,3,5-tris(aminophenyl)benzene (TAPB) were prepared by Kawagishi et al. [26]. Nguyen et al. [32] synthesized polyimide aerogels crosslinked with triisocyanates in one step at room temperature. In addition to different crosslinking agents, different structures of polyimide aerogels have been reported in the literature. Chen et al. [33] synthesized polyimide aerogels with carboxylic functionalization for CO_2_ capture. Moisture-resistant polyimide aerogels containing propylene oxide links were reportedin the backbone by Meador [34]. Guo [35] researched flexible polyimide aerogels with dodecane links. Wang [36] synthesized polyimide aerogels containing benzimidazole structures for oil–water separation. Cashman [37] synthesized flexible polyimide aerogels with neopentyl spacers in the backbone. Pantoja [38] synthesized polyimide aerogels containingaliphatic spacers in the polymer backbone. Therefore, it is highly valuable to design monomers with different molecular structures in the polymer backbone, which can endow polyimide aerogels with the appropriate functions to meet the needs of practical applications.

In this study, we successfully synthesized mesoporous polyimide aerogels with carboxyl from 2,2′-dimethyl-4,4′-diaminobiphenyl; 3,5-diaminobenzoic acid; pyromellitic dianhydride; 1,3,5-triaminophenoxybenzene. The repeat unit (*n*) is found to be 60 by using a ratio of *n* diamine units to (*n* + 1) dianhydride units to fabricate the oligomers. Using pyridine/acetic anhydride to catalyze imidization, polyimide wet gels are obtained by solvent exchange. Polyimide aerogel monoliths are acquired by supercritical CO_2_ drying. The microstructure and porous structure, as well as the morphology and the thermal stability of the aerogels is intensively investigated. Concurrently, we also examine the CO_2_ adsorption properties of polyimide aerogels with the carboxyl group.

## 2. Materials and Methods

### 2.1. Materials

1,3,5-tris(4-aminophenoxy)benzene (TAB) was synthesized according to the literature [39]. 2,2′-dimethyl-4,4′-diaminobiphenyl (DMBZ, CAS: 84-67-3), 3,5-diaminobenzoicacid (DABA, CAS: 535-87-5) and pyromellitic dianhydride (PMDA, CAS: 89-32-7) were obtained from Energy Chemical (Sarn Chemical Technology Co., Ltd., Shanghai, China). Grade *N*-methyl-2-pyrrolidinone (NMP, CAS: 872-50-4), grade pyridine (CAS: 110-86-1) and anhydrous ethanol were purchased from Sigma-Aldrich (Sigma Aldrich, Shanghai, China). Anhydrousacetic anhydride (Ac_2_O, CAS: 108-24-7) was obtained from Sinopharm Chemical Reagent Co., Ltd. (Sinopharm Chemical Reagent, Shanghai, China). All chemical reagents were used without further purification.

### 2.2. Preparation of TAB Cross-Linked Polyimide Aerogels

**Scheme 1:**
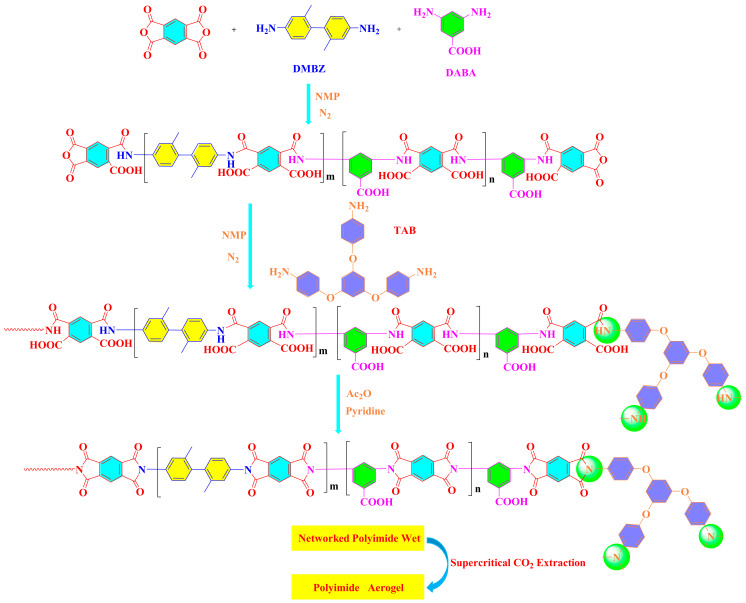
Synthesis route of polyimide aerogels with carboxyl.

Polyimide aerogels were prepared according to a modified protocol [40,41], and the procedure is shown in Scheme 1. Generally, polyimide aerogels are prepared from thepolyamic acid (PAA) oligomers end-capped by anhydride, crosslinked with cross-linking agent, and imidized using pyridine and acetic anhydride as the catalysts. In this work, the PAA oligomers were synthesized by reacting ndiamine (various DMBZ/DABA molar ratios) with (*n* + 1) dianhydride (PMDA) in NMP solvent, where *n* (*n* = 60) was the repeat units in the polyimide backbone, and the total weight of PAA oligomers in solution was formulated to be10 wt% in all cases. Asan example, the preparation of a polyimide aerogel with a DMBZ/DABA molar ratio of 50/50 was as follows: At low temperatures (0–10 °C), DABA (0.7608 g, 5 mmol) and 34 mL of NMP were added separately to a 100 mL one-necked flask equipped with a magnetic stirrer. After the diamine was completely dissolved, PMDA (2.2175 g, 10.16642 mmol) was added slowly to the above solution. The mixture was stirred until all the PMDA was dissolved, then DMBZ (1.0615 g, 5 mmol) was added. The solution was stirred until uniform, then a solution of TAB (0.0444 g, 0.1112 mmol) in 1 mL of NMP was added. The resulting solution was stirred continually for 10 min at room temperature, after which acetic anhydride (7.64 mL, 81.3312 mmol) and then pyridine (6.55 mL, 81.3312 mmol) were added. The solution was stirred vigorously for 5 min and poured into a cylindrical mold immediately. Gelation took place within 15 min. The gel was aged in the mold for one day. The solvent within the gel was then gradually exchanged to ethanol in 24 h intervals starting with 75% NMP in ethanol, followed by 50% NMP in ethanol, followed by 25% NMP in ethanol, and finally 100% ethanol. The ethanol was removed from the gel by supercritical CO_2_ extraction under 10 MPa at 50 °C followed by vacuum drying at 80 °C for 12 h to obtain polyimide aerogels.

### 2.3. Characterization

Attenuated total reflectance infrared spectroscopy (ATR-FTIR) of polyimide aerogels was performed on a Nicolet 8700 spectroscope (Thermo Fisher Scientific, Boston, MA, USA) with the range of 4000–400 cm^−1^ by averaging 32 scans. Fourier transform infrared (FTIR) spectra of polyimide aerogels and monomers were recorded using a Nicolet 8700 spectroscope with the range of 4000–400 cm^−1^ by averaging 32 scans. Samples were prepared by dispersing the complexes in KBr to form disks. X-ray photoelectron spectroscopy (XPS) analysis was performed on a Thermo Fisher K-Alpha (Thermo Fisher Scientific, Boston, MA, USA) employing an Al Kα (1486.68 eV) X-ray source equipped with a hemispherical analyzer. The microstructure of the polyimide aerogels was observed using field-emission scanning electron microscopy (FE-SEM) with a JSM-7610F Plus (HITACHI, Tokyo, Japan). Before measurement, the samples were bonded to conductive adhesive tapeto facilitate conduction. Surface area and pore volume analyses were performed on a BSD-PS1/2/4 analyzer (Bei Shi De, Beijing, China). Prior to measurements, the samples were degassed at 120 °C under high vacuum overnight. Adsorption and desorption isotherms of nitrogen were measured at −196.15 °C from 0 to 1.0 bar. The Brunauer–Emmett–Teller (BET) method was used to calculate the specific surface of thepolyimide aerogels. The pore size distribution was derived from Barret–Joyner–Halenda (BJH). Carbon dioxide adsorption isotherms were measured at 0 °C from 0 to 1.0 bar. Thermalgravimetric analysis (TGA) curves were recorded on a Q500 (TA Instruments, Newcastle, CA, USA) thermal analyzer by heating the samples (~10 mg) up to 800 °C with a ramping rate of 10 °C/min^−1^ under nitrogen flow.

## 3. Results and Discussion

### 3.1. Synthesis of Polyimide Aerogels and Characterization

**Figure 1:**
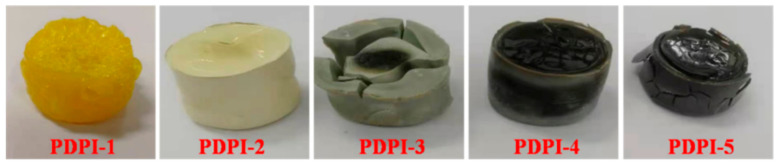
The polyimide aerogel monoliths.

As shown in Scheme 1, using NMP as a solvent, pyridine/Ac_2_O as a catalyst, and TAB as a crosslinker, polyimide aerogels with carboxyl were successfully prepared from PMDA and DMBZ/DABA by the co-polymerization method at room temperature. The molar ratios of DMBZ/DABA were 100/0, 75/25, 50/50, 25/75, and 0/100, and the corresponding polyimide aerogels were named PDPI-1, PDPI-2, PDPI-3, PDPI-4, and PDPI-5, respectively. PDPI-1~PDPI-5 samples were all monolithic as shown in Figure 1.

Although the PDPI-3–PDPI-5 samples were monolithic, they were brittle and could be turned into aerogel powder by gentle crushing, as shown in Figure 1. This may be due to the fact that the carboxyl content of polymer chains increased and the number of hydrogen bonds between molecular chains increased with the increase in DABA content, which led to the increase in rigidity and the decrease in toughness of polymer chains. However, the PDPI-1 and PDPI-2 samples had good toughness. Compared to previous porous materials, the synthesis of this aerogel is facile, the reaction conditions are mild, and the cost is low. The synthesis process uses NMP as the solvent, which has low toxicity, and environmentally friendly anhydrous ethanol is used for the solvent exchange. Therefore, the polyimide aerogel with carboxyl is suitable for large-scale production and application.

**Figure 2:**
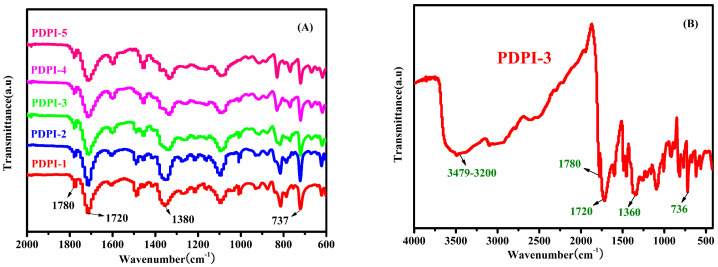
(**A**) ATR–FTIR spectra of polyimide aerogels and (**B**) FTIR spectrum of the PDPI-3 sample.

The microstructure of the polyimide aerogels was confirmed by ATR-FTIR, as shown in Figure 2A. As seen in the ATR-FTIR spectra, all samples exhibited typical characteristic imide peaks at 1780 cm^−1^ (imide C=O asymmetric stretching), 1720 cm^−1^ (imide C=O symmetric stretching), 1380 cm^−1^ (stretching bending vibration of the C–N–C moiety of the five-member imide ring), and 737 cm^−1^ (imide C=O bending vibration). However, the O–H absorption peak of the carboxyl was not found in the polymer spectrum, which was mainly due to the fact that the sensitivity of light at different wavelength intervals is not the same when the sample is detected by ATR-FTIR. The absorption peak intensity can increase due to the deep transmission of light in the sample at long wavelengths. The light transmission depth in the same sample is smaller at short wavelengths, and most of the light is reflected at the material surface. In order to illustrate this problem more clearly, we selected the PDPI-3 sample, which was relatively easy to grind into powder, and examined it with the FTIR instrument using the potassium bromide compression method, as shown in Figure 2B. It can be clearly observed that there was a broader OH absorption peak at 3479–3200 cm^−1^, which indicated that the carboxyl was not affected by the excess of acetic anhydride and pyridine. Furthermore, the amine and anhydride peaks of the monomers were not found in the spectrum. The typical asymmetric and symmetric stretching vibration absorption of the carbonyl group on the imide ring appeared at 1780 and 1720 cm^−1^. The other samples were not further tested because they had the same molecular chain structure as PDPI-3; only the molar amounts of monomers involved in polymerization were different. Apart from that, no absorption peaks were observed at 1800 and 1670 cm^−1^, indicating the absence of heteroimide ring structure in the structure. Therefore, the above information implies that full imidization of these polyimide aerogels occurs under the polymerization conditions [42,43].

**Figure 3:**
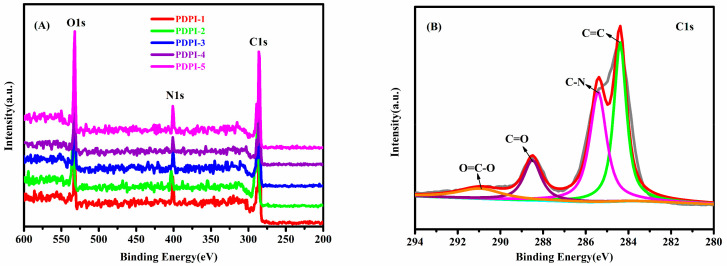

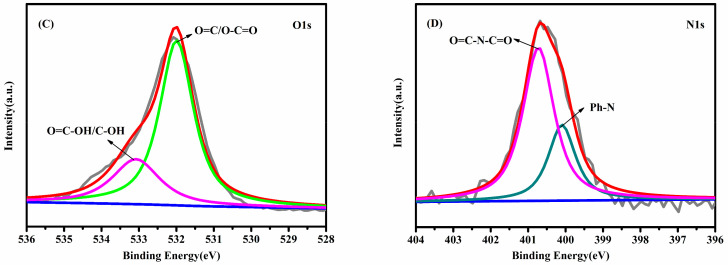
XPS full spectrum of all samples (**A**); (**B**) C1s, (**C**) O1s, and (**D**) N1s high-resolution XPS spectrum of PDPI-3.

X-ray photoelectron spectroscopy (XPS) study can reveal important information about functional groups. Thus, an XPS full spectrum for each sample is presented in Figure 3A. The spectra of all samples are identical and they all contain carbon, nitrogen, and oxygen elements. At the same time, there is no other impurity signal peak in the spectra. To obtain the bonding forms of these elements, we fitted the C1s/N1s/O1s spectra to split the peaks. The C1s deconvoluted spectrum of PDPI-3 (Figure 3B) displayed four different binding energies at 284.4, 285.4, 288.2, and 290.7 eV, which were ascribed to the C=C in the phenyl ring, C–N/O=C in the imide ring, and O=C–O in the carboxyl group, respectively [44]. Furthermore, the O1s spectrum of PTPI-3 exhibited two binding energy peaks (Figure 3C) at approximately 531.5 eV, corresponding to the C=O of the imide ring, and 533.7 eV was assigned to the C–OH of the carboxyl group [45]. In addition, the N1s spectrum of the PDPI-3 sample also showed two binding energy peaks (Figure 3D), which were located at 400.7 and 400.1 eV. The two binding energy peaks were very close to each other, and they both belonged to the C–N bond, where 400.1 eV is possibly attributed to Ph–N (where Ph refers to the phenyl ring) and 400.7 eV is possibly attributed to O=C–N–C=O [46,47]. The above information is sufficient to prove that the aerogel monolith structure contains an imide ring and a carboxyl group.

**Figure 4:**
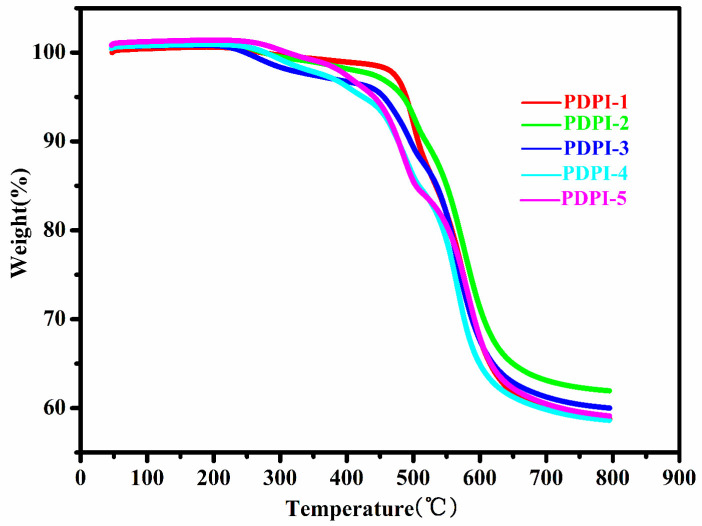
TGA curves of polyimide aerogels with carboxyl.

For each sample, the resultant aerogel solid could not dissolve in any solvent, suggesting a hyper crosslinked structure. The thermal stability of these resulting TAB crosslinked polyimide aerogels was evaluated by thermogravimetric analysis (TGA) under a nitrogen atmosphere from room temperature to 800 °C at a heat ramp of 10 °C/min. Detailed TGA curves and results are shown in Figure 4 and Table 1.

As shown in Figure 4, the thermal performance curves of all samples exhibited similar trends. There was a mass loss for PDPI-2–PDPI-5 at approximately 200–350 °C. This may have been caused by the decomposition of the carboxyl in DABA. The decomposition temperature of all polyimide aerogels was above 400 °C, which indicated that the aerogels have good temperature resistance. With an increase in DABA content, 5% thermal decomposition temperature (T_d_5%) decreased gradually and then slowly increased, as shown in Table 1. In the PDPI-1–PDPI-4 samples, the thermal decomposition temperatures decreased with the increase in DABA content. However, the thermal decomposition temperature of the PDPI-5 sample increased. This may have been caused by hydrogen bonding interactions between the polymer molecular chains.

### 3.2. Porous Structure and Surface Morphologies of Polyimide Aerogels

**Figure 5:**
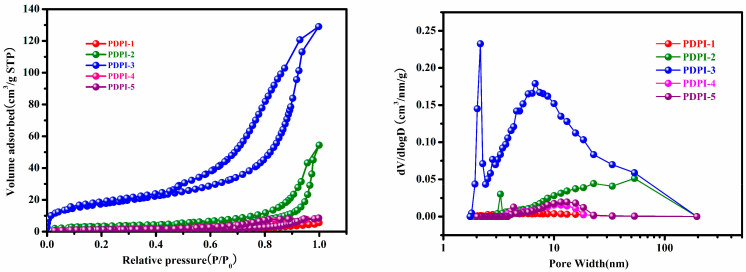
N_2_adsorption–desorption isotherms and pore size distributions of polyimide aerogels.

The surface area and pore volume of the polyimide aerogels were measured by nitrogen physical adsorption using the Brunauer–Emmett–Teller (BET) method. The porous parameters of polyimide aerogels are summarized in Table 2. Typical nitrogen adsorption–desorption isotherms at −196 °C are shown in Figure 5.

According to the IUPAC classification, it is obvious from Figure 5 that the PDPI-2 and PDPI-3 samples exhibited typical type IV adsorption–desorption isothermal curves, and the adsorption curves of the samples increased sharply in P/P_0_ = 0.4–1.0 bar, indicating the presence of mesopores in the polymer backbone. These mesopores may have mainly been from the pores formed by the aggregation of polymer nanofibers. At the same time, the adsorption–desorption curves of the PDPI-2 and PDPI-3 samples showed H2-type hysteresis loops. The hysteresis loop characteristic of the isotherm is associated with the capillary condensation of nitrogen in the mesopores [48] and the phenomena further indicated the existence of a large number of mesopores inside the PDPI-2 and PDPI-3 aerogels. According to the Barret–Joyner–Halenda (BJH) pore size analysis, it was shown that the PDPI-3 sample had narrow pore size distribution, as shown in Figure 5. It can be seen that the pore size distribution was in the range of 11.15~28.56 nm, as shown in Table 2, indicating the formation of mesopores in the polyimide aerogels according to IUPAC. The BET surface areas of the PDPI series samples were calculated and analyzed by the BET multipoint method, as shown in Table 2. Among all aerogels, the PDPI-3 sample showed the highest BET surface area of 62.03 m^2^/g, while the BET surface areas of the other samples were small. This may have been caused by the shrinkage that occurs during the preparation of aerogels and how the aerogel inevitably destroys the vesicles during the shrinkage process, which also leads to a reduced BET surface area. There are many additional factors affecting the shrinkage of aerogels [21], such as interactions between polymer chains and solvents, chains rigidities, and chain stacking density.

**Figure 6:**
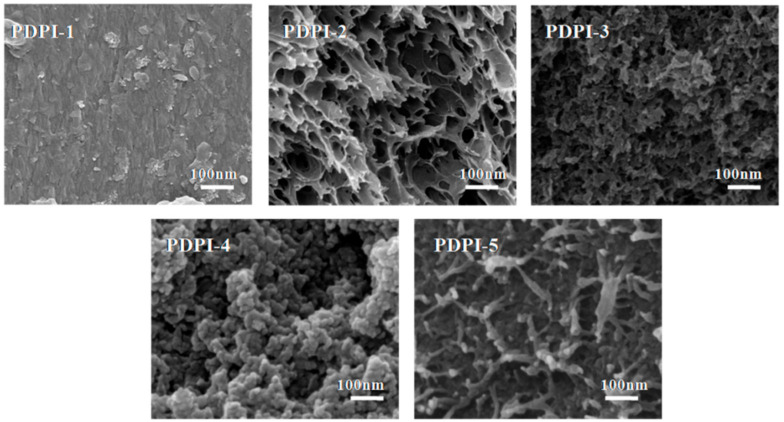
FE-SEM images of TAB crosslinked polyimide aerogels.

The surface morphologies of all aerogels were observed by FE-SEM, as shown in Figure 6.With the exception of the PDPI-1 sample, all other samples consisted of a three-dimensional network of tangled, fiber-like morphology with an irregular shape. This is similar to the morphologies of some reported polyimide aerogels [49]. The PDPI-1 sample was completely dense. The PDPI-4 and PDPI-5 polymer networks were relatively tightly stacked. The PDPI-2 and PDPI-3 samples were similar in morphology, but the pore size of PDPI-2 was significantly larger than that of PDPI-3. The morphologies of PDPI-2~PDPI-5 samples implied that there was a possible interpenetration of the network structure during the polyimide synthesis process since the polycondensations between diamine and dianhydride monomers are kinetically controlled, following the step polymerization mechanism.

### 3.3. CO_2_ Adsorption Property

**Figure 7:**
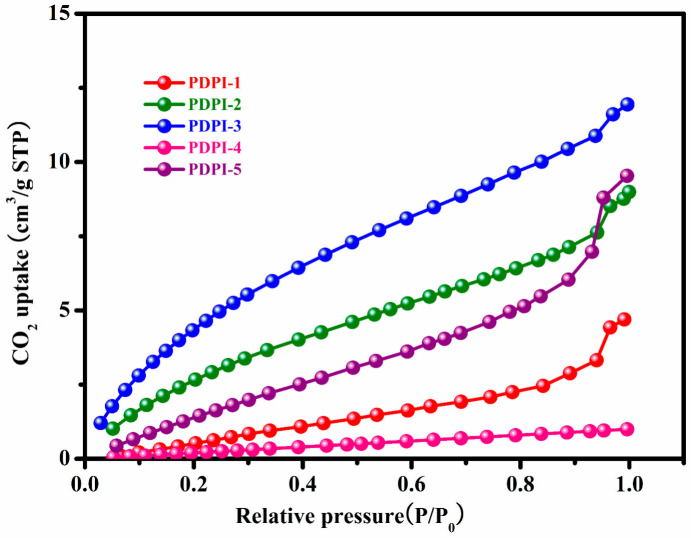
The CO_2_ uptake properties of the polyimide aerogels.

The CO_2_ uptake exhibited a rapid increase in the low-pressure region for the PDPI-3 sample, as shown in Figure 7, implying that CO_2_ molecules had favorable interactions with the porous polymer skeleton. It is clear that CO_2_ adsorption had not yet reached equilibrium when the pressure reached 1.0 bar, as shown in Figure 7, which means that the amount of CO_2_ adsorption would continue to increase if the pressure continued to rise. Compared to PDPI-3, the PDPI-4 sample had a smaller BET surface area. However, the CO_2_ adsorption capacity of PDPI-3 as 11.9 cm^3^/g, while the CO_2_ adsorption capacity of the PDPI-4 was 10.1 cm^3^/g. This difference in CO_2_ uptake is not substantial. This indicated that organic porous polymers with carboxyl functional groups can effectively enhance CO_2_ uptake [50,51]. This may be caused by dipole–quadrupole interactions between carboxyl and CO_2_ in the polyimide network [52,53]. Compared to other porous organic materials [54,55,56,57], the CO_2_ adsorption capacity of polyimide aerogels is relatively small, but they have a facile synthesis method and easy scale production, which are advantages with which other porous materials cannot compete. In this study, the highest CO_2_ uptake of polyimide aerogels was 11.9 cm^3^/g at 273 K and 1.0 bar. This value is also comparable to those of some porous materials with larger BET surface areas previously reported under the same conditions, such as porous polyimides PI2 (13.66 cm^3^/g) [52], PPBPI-PTC-CR (14.91 cm^3^/g) [58], mesoporous PIL (10.24 cm^3^/g) [59], and PyP (17.20 cm^3^/g) [60]. Therefore, the presence of DABA not only provides for the hydrogen bonding of the polymer, but also improves the adsorption capacity of carbon dioxide.

## 4. Conclusions

Polyimide aerogels were successfully prepared through the polycondensation of a new diamine with carboxyl (DABA), PMDA, and a TAB cross-linker. The resulting aerogel structures were confirmed by ATR-FTIR. We investigated the CO_2_ adsorption properties of samples PDPI-1–PDPI-5. We found that the introduction of carboxyl into the polyimide aerogels could significantly improve the CO_2_ uptake. The affinity possibly increased between the pore surface of the polyimide aerogels and the CO_2_ due to the existence of the carboxyl group. Therefore, the largest CO_2_ absorption capacity was 11.9 cm^3^/g for sample PDPI-3 at 273 K and 1.0 bar. Although the absorption capacity is not as high as those of microporous polyimide materials or reported COFs, the flexible processing and high yield make it possible to achieve large-scale production and application. Additionally, the facile and mild synthesis method can greatly reduce the cost of preparing materials. Thus, the above results showed that polyimide aerogels with carboxyl are promising candidates for CO_2_ capture in the field of environmental protection.

## Figures and Tables

**Table 1:** Thermal properties of polyimide aerogels.

Sample	T_d_5% (°C)
PDPI-1	489.36
PDPI-2	474.28
PDPI-3	440.69
PDPI-4	405.63
PDPI-5	413.56

**Table 2:** Porous parameters and CO_2_ uptake of polyimide aerogels.

Sample	S_BET_ (m^2^/g)	Vtotal (cm^3^/g)	Pore Size (nm)	CO_2_ Uptake (cm^3^/g)
PDPI-1	2.83	0.008	11.15	4.7
PDPI-2	10.91	0.078	28.56	9.0
PDPI-3	62.03	0.197	12.67	11.9
PDPI-4	3.47	0.013	14.52	10.1
PDPI-5	3.78	0.013	13.74	9.5

## Data Availability

The data presented in this study are available on request from the corresponding author.
